# Direct costs of prematurity and factors associated with birth and maternal conditions

**DOI:** 10.11606/s1518-8787.2022056003657

**Published:** 2022-06-07

**Authors:** Thamires Francelino Mendonça de Melo, Rodrigo Luiz Carregaro, Wildo Navegantes de Araújo, Everton Nunes da Silva, Aline Martins de Toledo

**Affiliations:** I Universidade de Brasília Faculdade de Ceilândia Programa de Pós-Graduação em Ciências da Reabilitação Brasília DF Brasil Universidade de Brasília. Faculdade de Ceilândia. Programa de Pós-Graduação em Ciências da Reabilitação. Brasília, DF, Brasil; II Universidade de Brasília. Faculdade de Ceilândia Brasília DF Brasil Universidade de Brasília. Faculdade de Ceilândia. Curso de Saúde Coletiva. Brasília, DF, Brasil

**Keywords:** Infant, Premature, Perinatal Care, economics, Costs and Cost Analysis, Intensive Care Units, Neonatal, Maternal and Child Health

## Abstract

**OBJECTIVE:**

To estimate the direct costs due to hospital care for extremely, moderate, and late preterm newborns, from the perspective of a public hospital in 2018. The second objective was to investigate whether factors associated with birth and maternal conditions explain the costs and length of hospital stay.

**METHODS:**

This is a cost-of-illness study, with data extracted from hospital admission authorization forms and medical records of a large public hospital in the Federal District, Brazil. The association of characteristics of preterm newborns and mothers with costs was estimated by linear regression with gamma distribution. In the analysis, the calculation of the parameters of the estimates (B), with a confidence interval of 95% (95%CI), was adopted. The uncertainty parameters were estimated by the 95% confidence interval and standard error using the Bootstrapping method, with 1,000 samples. Deterministic sensitivity analysis was performed, considering lower and upper limits of 95%CI in the variation of each cost component.

**RESULTS:**

A total of 147 preterm newborns were included. We verified an average cost of BRL 1,120 for late preterm infants, BRL 6,688 for moderate preterm infants, and BRL 17,395 for extremely preterm infants. We also observed that factors associated with the cost were gestational age (B = -123.00; 95%CI: -241.60 to -4.50); hospitalization in neonatal ICU (B = 6,932.70; 95%CI: 5,309.40–8,556.00), and number of prenatal consultations (B = -227.70; 95%CI: -403.30 to -52.00).

**CONCLUSIONS:**

We found a considerable direct cost resulting from the care of preterm newborns. Extreme prematurity showed a cost 15.5 times higher than late prematurity. We also verified that a greater number of prenatal consultations and gestational age were associated with a reduction in the costs of prematurity.

## INTRODUCTION

Preterm birth occurs at less than 37 weeks of pregnancy and is associated with maternal and neonatal conditions. According to the Brazilian Ministry of Health, prematurity has several causes, such as infections, smoking, absence of prenatal care, maternal age (under 14 and over 40 years of age), and obstetric or maternal complications (such as diabetes)^1^. Despite the high coverage of prenatal care in Brazil (private and public healthcare network), the effectiveness of this care is still low^2^, which may favor a high incidence of preterm births.

Preterm newborns require care due to the risk of complications, and prematurity is the factor that most influences the need for hospitalization^1,3^. Moreover, prematurity can impact both the neonatal period and childhood, increasing the demand for health care. Consequently, prematurity imposes increases in the spending of the health system^3^. In this sense, the economic impact mainly occurs due to the greater need for hospitalization^4^, immediate neonatal intensive care, with subsequent hospitalization in a neonatal intensive care unit^5^, in addition to long-term medical care^6^.

The evident economic impact caused by preterm birth has been demonstrated in studies in several countries^7^, which compared the costs between preterm birth and full-term birth. Late and moderate preterm newborns accounted for higher costs compared with full-term newborns in the first years of life. Overall, the costs are attributed to hospitalization, emergency, outpatient care, and prescription of medications^7^. Thus, the health system can be directly or indirectly affected by the health demands resulting from preterm birth.

However, although the costs of prematurity are known in the international context, there is a scarcity of information in the Brazilian context. Among the few studies found, we highlight Mwamakamba and Zucchi^10^, who estimated that the total direct costs of 84 preterm newborns of adolescent mothers were, in 2006, USD 195 thousand (BRL 1 million, in current values), which exponentially decreased with the increase in gestational age. Entringer et al.^11^ verified that the costs of the Kangaroo care unit were approximately BRL 5 million, and those of the conventional intermediate unit were BRL 7 million (25% higher than those of the Kangaroo care unit) in a hypothetical cohort of one thousand eligible newborns during hospitalization in these modalities. Regarding the cost of preterm and low-birth-weight newborns, Desgualdo et al.^5^ found that the average cost of hospitalization was USD 2,017, being higher in newborns with low birth weight. In addition, a difference was observed between the costs actually disbursed by the hospital and those reimbursed by the Brazilian Unified Health System (SUS), with reimbursement covering only 27.4% of the actual costs. These findings highlight the fact that prematurity generates a great economic burden in the context of health care^10^.

Estimating the costs of health care is relevant to guide rational decisions for allocating resources^12^, in addition to fostering a source of information to discuss and promote the development of public health policies. Investigating the influence of maternal and neonatal factors associated with prematurity in hospital costs can contribute to the understanding of how much this condition has impacted the SUS, and thus support and guide the public health policies in Brazil. Lastly, knowing the factors associated with preterm birth is important to guide future preventive strategies. Accordingly, we raise a question whether how much the country’s health system spends on hospital care for extremely, moderate, and late preterm infants. Moreover, if factors associated with birth and maternal conditions are associated with the cost and length of hospital stay.

Hence, the objectives of this study were to estimate the direct costs of hospital care for preterm newborns from the perspective of a public hospital in the Federal District of Brazil (DF), in 2018, and to investigate the influence of factors associated with birth and maternal conditions on these costs and length of hospital stay.

## METHOD

### Study Design

This is a cost-of-illness study, based on the data of payments charged in the *Autorização de Internação Hospitalar* (AIH *–* Hospital Admission Authorization form). For the analysis of associated factors, the information of the AIH form was complemented with information from the electronic medical records of mothers and newborns. The study was conducted in a large and secondary-care public hospital^13^, located in Ceilândia, the largest administrative region of the Federal District, with a population of approximately 432,927 inhabitants. Nevertheless, it also serves the population of the administrative region of Brazlândia (53,534 inhabitants), in addition to the surroundings of the Federal District, such as Águas Lindas de Goiás (217,698 inhabitants). The hospital has 275 inpatient beds distributed in neonatal, pediatric, obstetric, surgical, orthopedic, and internal medicine care, and eight beds of the neonatal intensive care unit (NICU). According to data from the establishment itself, in 2018 there were 6,508 deliveries.

The study was carried out from the perspective of SUS by the Ministry of Health, because our costs reflect the amounts reimbursed by it to hospital service providers, being of the top down type. The temporal horizon corresponded to one year (2018) and, therefore, no correction was required by inflation. Considering that the focus of the study was to investigate the costs and length of hospital stay related to newborns’ birth, the temporal horizon of one year was deemed adequate.

### Participants

The study population consisted of 147 preterm newborns born and hospitalized in the neonatology unit of the investigated hospital, which is composed of the NICU, conventional intermediate care and Kangaroo care units, and maternity/rooming-in. The newborns were classified according to gestational age for cost analysis: a) late preterm (34–36 weeks and six days of gestation); moderate preterm (28–34 weeks of gestation); and extremely preterm (< 28 weeks of gestation)^3^. The analysis of stratification by gestational age is necessary due to the greater need for care related to preterm infants with lower gestational age.

As exclusion criteria, the following were established: 1) presence of congenital malformations and genetic syndromes, considering that these conditions require greater assistance and they could be a bias to estimate costs; 2) newborns who died, as only the costs of hospitalization until hospital discharge were considered; 3) transfer to other healthcare units due to loss of follow-up.

Regarding the sample size, the study included all preterm newborns born in 2018 who had a record of NICU inpatient days and/or longer length of hospital stay (inpatient days in which the patient must remain hospitalized after the mean period considered for a specific hospital procedure)^14^ in their AIH forms. It is worth noting that in the investigated hospital, when the infant remained hospitalized in any neonatology unit, except for the NICU, the billing was identified in the AIH form by the longer length of hospital stay.

The study was approved by the Institutional Ethics Committee (FCE/SES, protocols no. 3.421.619 and no. 3.530.203).

### Variables

To describe the costs, the components described in the AIH forms of both hospital and professional services were analyzed. For hospital services, the following were considered: a) imaging tests; b) physiotherapeutic care; c) nutrition therapy; d) NICU inpatient day and longer length of hospital stay; e) treatment of diseases originating in the neonatal period and infectious diseases; f) others: kinetic-functional diagnosis, transfusions, cytopathologic tests, among others. As for professional services, the following were considered: consultations of healthcare professionals; treatment of diseases originating in the neonatal period; inpatient days (NICU and longer length of hospital stay).

For the characterization of newborns and association with costs and hospitalization time, the following variables were analyzed: gestational age (in weeks); birth weight (in grams); general classification of the newborn, categorized as: appropriate for gestational age (10–90^th^ percentile); small for gestational age (< 10^th^percentile); and large for gestational age (> 90^th^ percentile)^3^; classification of gestational age at birth, categorized as: late preterm; moderate preterm; and extremely preterm^3^; classification of birth weight, categorized as: low birth weight (< 2,500 g); very low birth weight (< 1,500 g); and extremely low birth weight (< 1,000 g)^3^; sex, categorized as: female or male; time of hospitalization in the NICU (days); need for hospitalization in the NICU, categorized as: yes or no; need for ventilatory support, considered as ventilatory support, mechanical ventilation, noninvasive ventilation, and oxygen therapy (oxygen HOOD, nasal catheter, and circulating oxygen in the incubator)^8^.

For the characterization of maternal variables and the association with the costs and time of hospitalization of newborns, maternal age (years), number of prenatal consultations (number of consultations), and gestational risk were analyzed. The latter was considered as: presence of urinary tract infection (categorized as yes or no); presence of gestational hypertension (categorized as yes or no); presence of gestational diabetes (categorized as yes or no); and type of delivery (cesarean or natural birth).

### Data Sources

Data for characterizing the sample and other variables were collected from electronic medical records available at the hospital, concerning preterm newborns in 2018. The cost data were extracted from the AIH forms, which were collected from the system of *Núcleo de Captação e Análise de Informações do SUS* (Center for Collecting and Analyzing Information of the SUS) of the hospital. The costs corresponded to the hospitalization period of preterm newborns, considering the period from birth to hospital discharge.

For the analysis of the total cost of each newborn, both the final value of the AIH form (which includes expenses with inpatient days, hospital and professional services) and the value of delivery (according to the values described in the *Sistema de Gerenciamento da Tabela de Procedimentos, Medicamentos, Órteses, Próteses e Materiais Especiais* [SIGTAP – Management System of the Table of Procedures, Medications, Orthotics, Prostheses and Special Materials] for each type of delivery: natural birth or cesarean) were considered. Thus, the value of delivery was included in the sum of the total costs, because it is a procedure related to birth and, consequently, it complements the objective of verifying the costs of prematurity. The costs were recorded in the Brazilian currency, *real* (BRL), and the length of stay was considered as the hospitalization stay (days).

For characterizing costs related to the hospitalization of preterm newborns, only the procedures that presented values approved in the AIH form, both hospital and professional services, were considered. The characterization of costs was described for each prematurity group: extremely, moderate, and late preterm infants.

### Data Analysis

The descriptive characterization of the costs was performed considering the stratification between hospital and professional services. In addition, the percentages of costs were separately described based on the total amount of hospital and professional services. The uncertainty of the cost parameters (BRL) was estimated by the 95% confidence interval (95%CI) and standard error (SE), using the Bootstrapping method, with 1,000 samples.

The characterization data of the participants were presented descriptively, estimating the mean and standard deviation for numerical variables and frequency measurements for categorical variables.

A deterministic sensitivity analysis was carried out, based on the lower and upper limits of the 95% confidence interval of each cost component (professional and hospital services), as parameters for estimates of variation in costs (total cost and total cost/newborn).

A generalized linear regression analysis with gamma distribution was performed. This model was adopted because it allows an analysis in which the response variables do not present normal distribution. Moreover, the model allows inferences about the average costs, which made it suitable for this study. For defining predictor variables, the collinearity between variables (established as r > 0.7) was verified. Collinear variables were excluded. Gestational age, birth weight, need for ventilatory support (mechanical ventilation, noninvasive ventilation, and oxygen therapy), and need for hospitalization in the NICU were considered as predictor variables related to the birth of preterm infants. As for predictor variables related to maternal conditions, maternal age, number of prenatal consultations, and the presence or not of gestational risks (hypertension, diabetes, and urinary infection) were considered. The response variables were hospital costs and hospitalization time.

The first regression analysis included the total cost of hospitalization as the response variable. Model 1 verified the impact of maternal variables (maternal age, number of prenatal consultations, presence of gestational hypertension, and urinary tract infection), and model 2 verified the impact of preterm newborns’ variables (use of mechanical ventilation, use of oxygen therapy, use of noninvasive ventilation, need for hospitalization in NICU, gestational age, and birth weight) on the total cost. The second regression analysis considered the length of hospital stay as the response variable. Model 3 verified the impacts of maternal variables (vide model 1); and model 4, the impact of newborns’ variables (vide model 2).

A 95% confidence interval (95%CI), with significance of 5% (p < 0.05) and identity link function for model estimation, was adopted. The Akaike Information Criterion was used to verify the models fit, confirming the gamma distribution as the most appropriate. The analysis was performed using the SPSS program, version 25.

## RESULTS

The medical records of all newborns in the investigated hospital with records of inpatient days in the NICU and/or longer length of hospital stay in 2018 were analyzed (totaling 372 eligible newborns). Of these, we excluded 187 full-term newborns and 38 preterm newborns based on the study criteria. Thus, we included 147 preterm newborns born in 2018.

The participants’ characterization data are presented in Table 1. A total of 51 NICU inpatient days were recorded and billed at AIH forms, and 119 longer length of hospital stays were recorded. The newborns who were not admitted to the NICU remained hospitalized in the other neonatology units of the hospital. In these cases, we could not identify in which specific unit of neonatology the newborn was hospitalized (these data are referenced in Table 1 as “other neonatology units”). It is also worth noting that we observed newborns who remained hospitalized both in the NICU and in the other neonatology units.

**Tabela 1:** Participants’ characterization data, represented in frequency of occurrence, mean, and standard deviation (when applicable).

Characteristics	n	Frequency (%)	Mean	SD
Gestational age (weeks)	147	-----	32.8	2.5
Birth weight (grams)	147	-----	1,792.0	526.0
General classification of newborns				
Appropriate for gestational age	98	66.7	-----	-----
Small for gestational age	48	32.7	-----	-----
Large for gestational age	1	0.7		
Classification of gestational age				
Extremely	4	2.7	25.7	1.3
Moderate	77	52.4	31.5	1.8
Late	66	44.9	34.8	0.8
Classification of birth weight				
Normal weight	15	10.2	2,834	296.3
Low birth weight	86	58.5	1,897.8	256.9
Very low birth weight	37	25.2	1,327.6	218.2
Extremely low birth weight	9	6.1	954.4	193.8
Sex				
Male	84	57.1	-----	-----
Female	62	42.2	-----	-----
Not identified	1	0.7	-----	
Hospitalization				
Only in the NICU	51	34.7	18.6^a^	2.1
NICU + other neonatology units	119	81	9.6^b^	7.8
Only in other neonatology units	96	65.3	8.94^c^	7.71
Ventilatory support				
Mechanical ventilation	37	25.2	-----	-----
Noninvasive ventilation^d^	90	61.2	-----	-----
Oxygen therapy	87	59.2	-----	-----
Maternal age (years)	147	------	26.3	7.4
Number of prenatal consultations	147	------	5.6	3.7
Gestational risk				
Diabetes	11	7.5	-----	-----
Gestational hypertension	31	21.1	-----	-----
Urinary tract infection	52	35.4	-----	-----
Type of delivery				
Natural birth	80	54.4	-----	-----
Cesarean	67	45.6	-----	-----

^a,b,c^ Mean (SD): mean and standard deviation on days of hospitalization: a) only in the neonatal intensive care unit (NICU); b) in NICU and in the other neonatology units (conventional intermediate care and Kangaroo care units and/or maternity/rooming-in); c) only in the other neonatology units described in b, except for the NICU.

^d^ Noninvasive ventilation: the infant can use more than one support modality.

Most of the infants were moderate preterm, with adequate weight for gestational age, and classified as low birth weight. The predominance was natural birth as type of delivery and most newborns were male. The mean gestational age was 32 weeks, with a mean weight of 1,792 grams. Most newborns required noninvasive ventilation support (Table 1).

The mean maternal age was 26.2 years and the mean number of prenatal consultations was 5.6 appointments. We observed that most mothers presented some gestational risk (Table 1).

The total costs due to birth and hospitalization of preterm newborns in 2018 were BRL 658,608.63. The average total cost per extremely, moderate, and late preterm newborn was BRL 17,395.46, BRL 6,688.92, and BRL 1,120.91, respectively. The average cost per extremely preterm newborn was 15.5 times and 2.6 times higher than late and moderate preterm newborns, respectively. The average cost per moderate preterm newborn was approximately 6 times higher than the late preterm infant (Table 2). The uncertainty of the cost parameters is presented in Table 3.

**Table 2:** Overview of expenses with components described in the AIH forms, separated by hospital and professional services, in addition to the value of delivery in each of the prematurity subgroups. The percentages of costs (%) were described in relation to the total value (hospital and professional services). Data collected at a public hospital in Brasília, DF, 2018.

Components	Extremely preterm (n = 4)		Moderate preterm (n = 77)		Late preterm (n = 66)		Total
	R$	%	R$	%	R$	%	
**Hospital services**							
Imaging tests	179.98	0.26	2,463.82	0.48	1,755.98	2.37	4,399.78
Physiotherapeutic care	3,352.16	4.82	21,598.71	4.19	2,347.07	3.17	27,297.94
Nutrition therapy	1,080.00	1.55	15,225.00	2.96	810.00	1.09	17,115.00
Inpatient days	49,310.40	70.87	352,772.43	68.49	19,375.49	26.19	421,458.32
Treatment of diseases originating in the neonatal period and infectious diseases	2,279.34	3.28	31,604.35	6.14	19,023.29	25.71	52,906.98
Other	1,735.85	2.49	3,833.74	0.74	826.82	1.12	6,396.41
Cost of delivery	1,300.33	1.87	26,803.22	5.20	2,3887.42	32.29	51,990.97
Total concerning hospital services	59,238.06	85.13	454,301.27	88.21	68,026.07	91.95	581,565.40
**Professional services**							
Medical consultations	198.52	0.29	5,225.70	1.01	3,074.91	4.16	8,499.13
Treatment of diseases originating in the neonatal period	2,009.27	2.89	1,415.25	0.27	1,184.18	1.60	4,608.70
Inpatient days	8,136.00	11.69	54,104.40	10.50	1,695.00	2.29	63,935.40
Total concerning professional services	10,343.79	14.87	60,745.35	11.79	5,954.09	8.05	77,043.23
**Total costs**	**69,581.85**	**100.00**	**515,046.62**	**100.00**	**73,980.16**	**100.00**	**658,608.63**

AIH: hospital admission authorization form.

**Table 3:** Uncertainty analysis of cost parameters (BRL). Estimates of 95% confidence interval and standard error were performed using the Bootstrapping method, with 1,000 samples.

	Mean	SE	95%CI	
			Lower limit	Upper limit
**Hospital services**				
Imaging tests	29.93	2.64	24.72	35.02
Physiotherapeutic care	185.70	32.47	127.22	250.18
Nutrition therapy	116.42	14.04	89.79	144.88
Inpatient days	2,867.06	411.62	2,097.51	3,677.69
Treatment of diseases originating in the neonatal period and infectious diseases	359.91	19.20	322.46	397.51
Other	41.61	11.19	21.85	65.63
Cost of delivery	353.68	5.77	342.33	365.02
**Total concerning hospital services**	**3,954.33**	**460.21**	**3,084.50**	**4,887.49**
**Professional services**				
Medical consultations	58.33	2.54	53.50	63.25
Treatment of diseases originating in the neonatal period	18.10	1.20	16.23	20.70
Inpatient days	436.77	67.80	306.32	569.55
**Total concerning professional services**	**513.22**	**68.15**	**382.48**	**647.81**
**Total costs**	**4,467.55**	**527.98**	**3,469.43**	**5,537.15**

SE: standard error; 95%CI: 95% confidence interval.

Expenses with hospital services were 7.5 times higher than professional services. More than 70% of the total expenses corresponded to inpatient days (sum of the values of inpatient days corresponding to hospital and professional services). The expenses with treatment of diseases originating in the neonatal period and infectious diseases, expenses with delivery, and physiotherapeutic care corresponded to 8.7%, 7.9%, and 4.14% of total expenses, respectively (Table 2).

The number of NICU inpatient days in extremely, moderate, and late preterm infants was 120 (4 newborns), 849 (77 newborns), and 44 (66 newborns), respectively. These inpatient days totaled an average cost per newborn of BRL 14,361.30 (extremely preterm), BRL 5,284.11 (moderate preterm), and BRL 319.92 (late preterm). Data on regression are presented in Table 4.

**Table 4:** Data on the association between factors related to birth and maternal conditions and the cost and length of hospital stay. Data collected at a public hospital in Brasília, DF, 2018.

Variables	Total cost (BRL)			Length of hospital stay (days)		
	B^b^	95%CI	p	B^b^	95%CI	p
**Birth-related variables**						
**Constant**	5,624.40	1,782.10 to 9,466.60	-	87.21	66.79 to 107.63	-
Gestational age (weeks)	-123.00	-241.60 to -4.50	0.04	-2.20	-2.78 to -1.61	< 0.01
Birth weight (grams)	-0.20	-0.50 to 0.03	0.08	-0.03	-0.004 to -0.002	< 0.01
Admission to the NICU (yes or no)^a^	6,932.70	5,309.40 to 8,556.00	< 0.01	1.67	-1.52 to 4.87	0.30
Use of mechanical ventilation (yes or no)^a^	736.90	-387.60 to 1,861.50	0.20	0.50	-3.52 to 4.51	0.81
Use of noninvasive ventilation (yes or no)^a^	91.20	-156.90 to 339.40	0.47	3.04	1.14 to 4.92	< 0.01
Oxygen therapy (yes or no)^a^	21.40	-216.20 to 258.90	0.86	1.43	0.134 to 2.718	0.03
**Maternal variables**						
**Constant**	7,771.40	3,588.20 to 11,954.60	-	22.20	14.36 to 30.09	-
Maternal age (in years)	-62.70	-190.20 to 64.80	0.33	-0.20	-0.48 to 0.02	0.07
Prenatal consultations (number)	-227.70	-403.30 to -52.00	0.01	-0.51	-1.13 to 0.12	0.11
Gestational hypertension (yes or no)^a^	1,820.00	-1,375.40 to 5,015.50	0.26	6.36	-0.16 to 12.88	0.06
Urinary tract infection (yes or no)^a^	-1,429.50	-3,444.40 to 585.30	0.16	0.14	-5.62 to 5.88	0.96

95%CI: 95% confidence interval; NICU: neonatal intensive care unit.

^a^ Absence of the condition (not) was considered a reference category.

^b^ Parameter estimation.

We verified an association between maternal variables and costs. Each prenatal consultation was associated with a reduction of BRL 227.69 in the total cost (Table 3). We found an association between costs and variables related to birth, in which gestational age was associated with a reduction of BRL 123.04 in total cost. Furthermore, the need for hospitalization in the NICU was associated with an increase of BRL 6,932.70 in the total cost (Table 4).

Regarding the length of hospital stay, we observed that gestational hypertension was associated with an increase of six days in the length of stay (Table 4). As for the variables related to birth, the higher the gestational age, the shorter the length of stay (Table 4). The use of noninvasive ventilation was associated with an increase of three days of hospitalization and one day of oxygen therapy use. Birth weight significantly contributed to the model.

**Figure 1:**
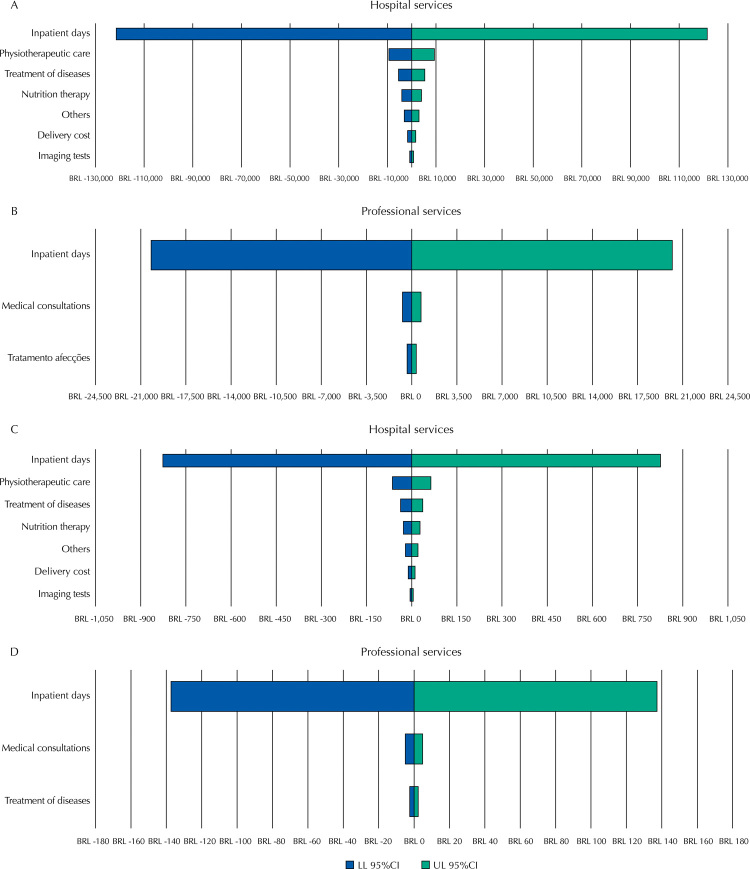
Sensitivity analysis considering the variations in parameters (lower and upper limits of the 95% confidence interval) of each cost component (professional and hospital services), considering the total costs (A and B) and total costs/newborn (C and D).

According to the sensitivity analysis, we identified that the total costs and total costs associated with hospital inpatient days, inpatient days of professional services, and physiotherapeutic care were the components that most influenced the model (Figure 1), indicating variations of approximately 18%, 3%, and 1.4%, respectively.

LL 95%CI: lower limit of 95% confidence interval; UL 95%CI: upper limit of 95% confidence interval.

## DISCUSSION

The present study demonstrated an average cost of BRL 1,120 for late preterm infants, BRL 6,688 for moderate preterm infants, and BRL 17,395 for extremely preterm infants admitted to a public hospital in 2018. In addition, the factors gestational age, hospitalization in NICU, and number of prenatal consultations were associated with the total cost. Furthermore, the factors presence of gestational hypertension, gestational age, use of noninvasive ventilation, and use of oxygen therapy were associated with length of hospital stay.

The total costs of preterm birth were approximately BRL 658 thousand. This amount considered expenses of only eight NICU beds that are billed at the investigated hospital. We found that the greatest expense when considering the components described in the AIH form was related to hospital inpatient days, which comprise all the actions necessary for life maintenance, including professional assistance for 24 uninterrupted hours. When analyzing the costs of each group of newborns, we observed that extremely preterm newborns have an average cost per infant of approximately 3 times the moderate preterm infants and 15 times the late preterm infants.

Gestational age and birth weight were important factors for costs and length of hospital stay. Accordingly, hospital costs were higher in newborns with lower gestational age. The increase in each week of gestational age was associated with a decrease in costs and hospitalization time, while the increase of each gram of birth weight was associated with a decrease in hospitalization time. These findings are corroborated by a systematic review^15^. Due to physiological immaturity, preterm newborns are in need of ventilatory support, feeding aid, and temperature regulation, being hospitalized until they reach weight and discharge criteria^8^. These aspects contribute to the increase in hospitalization time and, consequently, to greater exposure to stressors that impact the newborn’s development^16^. Such aspects may explain the higher cost with the inpatient days of extremely preterm newborns.

Regarding the clinical conditions of the newborns, we observed that the need for NICU hospitalization led to an increase in costs. The need for noninvasive ventilation and oxygen therapy were associated with increased length of hospital stay. The need for NICU hospitalization was associated with an increase of approximately BRL 6 thousand in the total cost. Previous studies have also demonstrated high costs with prematurity, from birth to NICU hospitalization^5,9,17,18^. These findings corroborate our study and could be explained by the type of support and resources offered, such as inpatient days, tests, medications, oxygen, and professional fees.

Noninvasive ventilation was associated with a 3-day increase in hospitalization, while the use of oxygen therapy led to an increase of 1 day. These findings may be justified by frequent respiratory conditions or obstructive pulmonary diseases in preterm newborns^8,18,19^. Studies have shown that these conditions, both in neonatal hospitalization and after hospital discharge, as well as in the first years of life, result in costs associated with newborns’ hospitalization and outpatient follow-up^8,19^. Infants with respiratory conditions may require home oxygen therapy or, during seizures, follow-up and medication. Moreover, a previous study showed that children with respiratory distress syndrome had higher costs compared with children without the syndrome^20^, and that the cost of respiratory conditions represented 25% of the total cost of hospitalizations of preterm newborns^21^.

The presence of ventilatory support was not associated with increases in the cost of hospitalization. Nonetheless, we consider relevant the fact that preterm newborns present pulmonary immaturity and, at birth, they are exposed to factors that can trigger injuries, including mechanical ventilation^22^. Weaning from ventilation is slow and prolongs hospitalization, because it depends on the resolution of lung injury or other conditions^23^. Thus, the use of mechanical ventilation influences hospital stay and, consequently, it should increase costs. However, we assume that the costs were not impacted, as the reference values of this procedure, according to SIGTAP, are lower than those charged in private health care. Therefore, changes in the perspective of the study could alter the costs.

We also demonstrated that each additional prenatal consultation was associated with a cost reduction. This reduction may be related to the reduction of perinatal complications as long as prenatal care is adequately taken^24,25^. This datum is relevant, because the absence of prenatal follow-up may increase the risk of prematurity by up to 2.8 times^26^. In Brazil, several studies have demonstrated an inversely proportional relationship between prematurity and prenatal care^24,25^. Prenatal care involves several health prevention and promotion actions, diagnosis and treatment, structured in at least six consultations, one in the first trimester of pregnancy, two in the second trimester, and three in the third trimester^24^. Our data emphasize the importance of prenatal care, considering that the costs of care of newborns of mothers who did not receive prenatal care may be higher when compared with newborns with prenatal care^27^. Prenatal follow-up is also essential for providing care to mothers with gestational hypertension (risk factor for prematurity and gestational complications). Gestational hypertension can cause intrauterine growth restriction, fetal distress, low birth weight, and prematurity^28^. We found that gestational hypertension was associated with a 6-day increase in hospitalization. These results might be explained by the risk factor for fetal complications imposed by this condition, which can cause anoxia^29^ and, consequently, require respiratory support. Gagliardi et al.^30^ also found a positive association between gestational hypertension and the risk of acute and chronic respiratory problems in newborns.

We verified an association between interventions and conditions related to prematurity in the hospital context. It is worth addressing the strengthening of primary health care, which can help to reduce hospital care costs in the context of prematurity. Previous studies have shown that the absence of prenatal follow-up is a risk factor for preterm birth^24,25,27^. In addition, we found an association between prenatal consultations and the increased cost of preterm newborns. Thus, we assume that the increase in coverage and expansion of actions to involve pregnant women in prenatal care could prevent prematurity and contribute to the reduction of the economic impact of this condition.

Our study considered only the SUS perspective. However, we found that the investigated hospital performed procedures that were not computed, for instance, hospitalization in neonatal intermediate care units. These units are not accredited by the Ministry of Health and, thus, the costs were not account for in this research. Nevertheless, the health care provided to preterm newborns in these services was considered. Hence, we assume that the costs of prematurity might be higher. This is a relevant finding, because the accreditation of the neonatal intermediate care units of the investigated hospital could expand the government’s economic resources and fund transfers. Finally, to consider the costs of prematurity from the perspective of the investigated hospital, we found that the direct costs due to hospital care for preterm newborns was considerable, as the average cost of hospitalization of a preterm newborn was BRL 4,480.33, while the average cost of pediatric hospitalizations, considering the treatment of chronic lower respiratory disease (the most prevalent disease in the hospital), was BRL 548.03.

The sensitivity analysis showed that the value of inpatient days of hospital services and professional services was the component that most influenced the total cost estimated by our study. Such findings indicate that inpatient days are important motivators of the model and present a greater determination of total costs. This was expected, considering that ICU inpatient days are more expensive and, additionally, this sector demands specialized care with greater complexity^31^. The sensitivity analysis data were based on variation that occurred within each AIH form analyzed in the study, which reflects the reality of recording the procedures used by preterm newborns of a public hospital. Hence, it was not based on arbitrary assumptions. Although there may be underreporting of performed procedures, we noticed no substantial variation in the amounts reimbursed by the Ministry of Health to hospitals for the investigated population. This aspect was demonstrated by univariate sensitivity analyses, in which the parameter of hospitalization days determined a variation of 18% in the estimated total cost.

Our study has limitations, including the reduced number of participants, because, although the investigated hospital has a high rate of deliveries, it does not have records to identify all preterm newborns. All preterm newborns who billed inpatient days were included; however, the sample did not include all preterm infants born in the hospital as the intermediate care units of the hospital are not accredited by the Ministry of Health and, consequently, do not generate a record of neonatal inpatient days in the AIH form. Therefore, preterm infants who remained hospitalized only in these units and did not generate hospitalization costs were not included in our study. Thus, we sought to minimize bias by including all available data on costs regarding the hospitalization of preterm newborns.

## CONCLUSION

Our findings showed that the direct costs due to hospital care for preterm newborns in a large hospital in 2018 was BRL 658,608. We verified that extreme prematurity imposed a cost 15.5 times higher compared with late prematurity. In addition, we observed that a greater number of prenatal consultations and gestational age were associated with a reduction in the costs of prematurity.
