# Supplementary material for: Direct costs of prematurity and factors associated with birth and maternal conditions
Source: Rev Saude Publica. 2022 Jun 7;56:49. doi: 10.11606/s1518-8787.2022056003657 (PMC9239337; doi:10.11606/s1518-8787.2022056003657)
Supplement: Translation

# Translation: Custos diretos da prematuridade e fatores associados ao nascimento e condições maternas

**Authors:** Thamires Francelino Mendonça de Melo, Rodrigo Luiz Carregaro, Wildo Navegantes de Araújo, Everton Nunes da Silva, Aline Martins de Toledo

**Affiliations:** I Brasil Universidade de Brasília. Faculdade de Ceilândia. Programa de Pós-Graduação em Ciências da Reabilitação. Brasília, DF, Brasil; II Brasil Universidade de Brasília. Faculdade de Ceilândia. Curso de Saúde Coletiva. Brasília, DF, Brasil

**Keywords:** Recém-Nascido Prematuro, Assistência Perinatal, economia, Custos e Análise de Custo, Unidades de Terapia Intensiva Neonatal, Saúde Materno-Infantil

## Abstract

**OBJETIVOS:**

Estimar os custos diretos advindos com a assistência hospitalar a recém-nascidos prematuros extremos, moderados e tardios, sob a perspectiva de um hospital público em 2018. O segundo objetivo foi investigar se fatores associados ao nascimento e às condições maternas explicam os custos e o tempo de permanência hospitalar.

**MÉTODOS:**

Estudo de custo da doença, com extração de dados a partir das autorizações de internação hospitalares e prontuários de um hospital público de grande porte do Distrito Federal. Estimou-se a associação de características dos recém-nascidos prematuros e das genitoras nos custos por meio de regressão linear com distribuição *gamma*. Na análise, adotou-se o cálculo dos parâmetros das estimativas (B), com intervalo de confiança de 95% (IC95%). Os parâmetros de incerteza foram estimados pelo intervalo de confiança de 95% e erro padrão por meio do método de *Bootstrapping*, com 1.000 amostragens. Realizou-se análise de sensibilidade determinística, considerando limites inferiores e superiores do IC95% na variação de cada componente de custo.

**RESULTADOS:**

Foram incluídos 147 recém-nascidos prematuros. Verificamos um custo médio de R$ 1.120 para prematuros tardios, R$ 6.688 para prematuros moderados e R$ 17.395 para prematuros extremos. Verificamos também que os fatores associados ao custo foram idade gestacional (B = -123,00; IC95% -241,60 a -4,50); internação em UTI neonatal (B = 6.932,70; IC95% 5.309,40–8.556,00) e número de consultas pré-natal (B = -227,70; IC95% -403,30 a -52,00).

**CONCLUSÕES:**

Verificamos um custo direto considerável advindo da assistência a recém-nascidos prematuros. A prematuridade extrema demonstrou um custo 15,5 vezes maior comparado à tardia. Verificamos ainda que uma maior quantidade de consultas pré-natal e a idade gestacional foram associadas a uma redução dos custos da prematuridade.

## INTRODUÇÃO

O nascimento prematuro ocorre com menos de 37 semanas de idade gestacional e está associado a condições maternas e neonatais. Segundo o Ministério da Saúde, a prematuridade possui diversas causas, como infecções, tabagismo, ausência de cuidado pré-natal, idade materna (menor de 14 e maior de 40 anos) e complicações obstétricas ou maternas (como diabetes)^1^. Apesar da alta cobertura da assistência pré-natal no Brasil (rede privada e pública), a efetividade dessa assistência ainda é baixa^2^, o que pode favorecer uma alta incidência de partos prematuros.

Recém-nascidos prematuros demandam cuidados devido ao risco de complicações, sendo a prematuridade o fator que mais influencia a necessidade de internação^1,3^. Adicionalmente, a prematuridade pode gerar impactos tanto no período neonatal quanto na infância, aumentando a demanda por cuidados em saúde. Consequentemente, a prematuridade impõe aumentos nos gastos do sistema de saúde^3^. Nesse sentido, o impacto econômico ocorre, principalmente, pela maior necessidade de internação^4^, cuidados intensivos neonatais imediatos, com subsequente internação em unidade de terapia intensiva neonatal^5^, além da assistência médica a longo prazo^6^.

O evidente impacto econômico causado pelo nascimento prematuro tem sido demonstrado em estudos de diversos países^7^, os quais compararam os custos entre o nascimento prematuro e o nascimento a termo. Recém-nascidos prematuros tardios e moderados acarretaram, nos primeiros anos de vida, custos maiores comparados aos recém-nascidos a termo. Em geral, os custos são atribuídos à hospitalização, emergência, atendimentos ambulatoriais e prescrição de medicamentos^7^. Deste modo, verifica-se que o sistema de saúde pode ser afetado direta ou indiretamente pelas demandas em saúde advindas do nascimento prematuro.

Entretanto, apesar dos custos com a prematuridade serem conhecidos no contexto internacional, há escassez de informações no contexto brasileiro. Entre os poucos estudos encontrados, destacamos Mwamakamba e Zucchi^10^, que estimaram que os custos diretos totais de 84 recém-nascidos prematuros de mães adolescentes foram, em 2006, de US$ 195 mil (R$ 1 milhão, em valores atualizados), os quais diminuíram exponencialmente com o aumento da idade gestacional. Entringer et al.^11^ verificaram que os custos da unidade Canguru foram de aproximadamente R$ 5 milhões, e os da unidade intermediária convencional R$ 7 milhões (25% superior às da unidade Canguru) em uma coorte hipotética de mil recém-nascidos elegíveis durante a internação nessas modalidades. Quanto ao custo do recém-nascido prematuro e de baixo peso ao nascer, Desgualdo et al.^5^ verificaram que o custo médio da hospitalizacão foi de US$ 2.017, sendo maior nos recém-nascidos com menor peso ao nascer. Além disso, observou-se uma diferença entre os custos efetivamente desembolsados pelo hospital e os reembolsados pelo Sistema Único de Saúde (SUS), com o reembolso cobrindo apenas 27,4% dos custos reais. Tais achados reforçam o fato de que a prematuridade gera um grande ônus econômico no âmbito da assistência à saúde^10^.

Estimar os custos da atenção à saúde é relevante para nortear decisões racionais para a alocação de recursos^12^, além de fomentar uma fonte de informação para discutir e propiciar o desenvolvimento de políticas públicas de saúde. Investigar a influência de fatores maternos e neonatais associados à prematuridade nos custos hospitalares pode contribuir com a compreensão do quanto essa condição tem impactado o SUS, e, assim, subsidiar e orientar as políticas públicas de saúde vigentes no Brasil. Por fim, conhecer os fatores relacionados ao nascimento pré-termo é importante para nortear futuras estratégias preventivas. Nesse sentido, levanta-se uma pergunta sobre quanto o sistema de saúde do país gasta com a assistência hospitalar de crianças nascidas prematuras extrema, moderada e tardias. Ademais, se fatores associados ao nascimento e às condições maternas estão associados ao custo e tempo de permanência hospitalar.

Deste modo, os objetivos deste estudo foram estimar os custos diretos da assistência hospitalar a recém-nascidos prematuros sob a perspectiva de um hospital público do Distrito Federal (DF) em 2018 e investigar a influência de fatores associados ao nascimento e às condições maternas nesses custos e no tempo de permanência hospitalar.

## MÉTODO

### Delineamento

Trata-se de um estudo de custo da doença, realizado com base nos dados de pagamentos lançados na autorização de internação hospitalar (AIH). Para análise de fatores associados, as informações das AIH foram complementadas com informações dos prontuários eletrônicos das mães e dos recém-nascidos. O estudo foi realizado em hospital público de atenção secundária e grande porte^13^, localizado em Ceilândia, a maior região administrativa do DF, com uma população aproximada de 432.927 habitantes. No entanto, ele também atende a população da região administrativa de Brazlândia (53.534 habitantes), além do entorno do DF, como Águas Lindas de Goiás (217.698 habitantes). O hospital possui 275 leitos de internação distribuídos em atendimentos neonatais, pediátricos, obstétricos, cirúrgicos, ortopédicos e de clínica médica, e 8 leitos de unidade de terapia intensiva neonatal (UTIN). Conforme dados do próprio estabelecimento, em 2018 ocorreram 6.508 partos.

O estudo foi realizado na perspectiva do SUS por meio do Ministério da Saúde, pois nossos custos refletem os valores reembolsados por ele aos provedores de serviços hospitalares, sendo do tipo *top down* (macrocusteio). O horizonte temporal correspondeu a um ano (2018) e, por este motivo, não foi necessária correção pela inflação. Tendo em vista que o foco do estudo foi investigar os custos e o tempo de permanência relacionados ao nascimento dos recém-nascidos, o horizonte temporal de 1 ano foi considerado adequado.

### Participantes

A população foi composta por 147 recém-nascidos prematuros nascidos e internados na unidade de neonatologia do hospital investigado, que é composta pela UTIN, unidades de cuidados intermediários convencional e Canguru e maternidade/alojamento conjunto. Os recém-nascidos foram classificados de acordo com a idade gestacional para a análise dos custos: a) prematuro tardio (34–36 semanas e seis dias de gestação); prematuro moderado (28–34 semanas de gestação) e prematuro extremo (< 28 semanas de gestação)^3^. A análise da estratificação pela idade gestacional se faz necessária devido a maior necessidade de cuidados relacionados aos prematuros com menor idade gestacional.

Como critério de exclusão, se estabeleceu: 1) presença de más-formações congênitas e síndromes genéticas, tendo em vista que essas condições demandam maior assistência e que elas poderiam ser um viés para a estimativa dos custos; 2) recém-nascidos que foram a óbito, pois foi considerado apenas os custos da internação até a alta hospitalar; 3) transferência a outras unidades de saúde devido à perda de seguimento.

Em relação ao tamanho amostral, foram incluídos todos os recém-nascidos prematuros nascidos em 2018 que possuíam registro de diárias de UTIN e/ou diárias de permanência a maior (diárias em que o paciente necessita permanecer internado após o período médio referido para um procedimento hospitalar específico)^14^ em suas AIH. Destaca-se que no hospital investigado, quando o lactente permanecia internado em qualquer unidade da neonatologia, à exceção da UTIN, o faturamento era identificado nas AIH por meio de diárias de permanência a maior.

O estudo foi aprovado pelo Comitê de Ética Institucional (FCE/SES, protocolos nº 3.421.619 e nº 3.530.203).

### Variáveis

Para descrição dos custos foram analisados os componentes descritos nas AIH tanto dos serviços hospitalares quanto profissionais. Para os serviços hospitalares, foram considerados: a) exames de imagem; b) assistência fisioterapêutica; c) terapia nutricional; d) diária de UTIN e de permanência a maior; e) tratamento de afecções originadas no período neonatal e doenças infecciosas; f) outros: diagnóstico cinético-funcional, transfusões, exames cito patológicos, dentre outros. Em relação aos serviços profissionais, foram consideradas as consultas de profissionais de saúde; tratamento de afecções originadas no período neonatal; diárias (UTIN e de permanência a maior).

Para caracterização dos recém-nascidos e associação com os custos e tempo de internação, foram analisadas as variáveis: idade gestacional (em semanas); peso ao nascer (em gramas); classificação geral do recém-nascido, categorizada em: adequado para idade gestacional (percentil 10–90); pequeno para idade gestacional (percentil < 10); e grande para idade gestacional (percentil > 90)^3^; classificação da idade gestacional ao nascer, categorizada em: prematuro tardio; prematuro moderado e prematuro extremo^3^; classificação do peso ao nascer, categorizada em: baixo peso (< 2.500 g); muito baixo peso (< 1.500 g) e extremo baixo peso (< 1.000 g)^3^; sexo, categorizado em: feminino ou masculino; tempo de internação na UTIN (dias); necessidade de internação na UTIN, categorizado em: sim ou não; necessidade de suporte ventilatório, considerado como suporte ventilatório a ventilação mecânica, a ventilação não-invasiva e a oxigenioterapia (HOOD, cateter nasal e oxigênio circulante na incubadora)^8^.

Para a caracterização das variáveis maternas e da associação com os custos e tempo de internação dos recém-nascidos, foram analisados a idade materna (anos), a quantidade de consultas de pré-natal (número de consultas) e o risco gestacional, considerado como: presença de infecção do trato urinário (categorizado em sim ou não); presença de síndrome hipertensiva (categorizado em sim ou não); presença de diabetes gestacional (categorizado em sim ou não); e tipo de parto (cesárea ou normal).

### Fontes de Dados

Os dados para caracterização da amostra e demais variáveis foram coletados por meio dos prontuários eletrônicos disponíveis no hospital, sendo correspondente aos recém-nascidos prematuros no ano de 2018. Os dados de custo foram extraídos das AIH, as quais foram levantadas por meio do sistema do Núcleo de Captação e Análise de Informações do SUS do hospital. Os custos corresponderam ao período de internação dos recém-nascidos prematuros, considerando o período desde o nascimento até a alta hospitalar.

Considerou-se, para análise do custo total de cada recém-nascido, tanto o valor final da AIH (que envolve gastos com diárias, serviços hospitalares e profissionais) quanto o valor do parto (considerando-se o valor que consta no Sistema de Gerenciamento da Tabela de Procedimentos, Medicamentos, Órteses, Próteses e Materiais Especiais (SIGTAP) para cada tipo de parto: normal ou cesárea). Deste modo, o valor do parto foi incluído no somatório do total de custos, pois compõe um procedimento relacionado ao nascimento e, consequentemente, complementa o objetivo de verificar os custos da prematuridade. Os custos foram contabilizados em reais (R$) e o tempo de permanência foi considerado pelo período de permanência no hospital (dias).

Para a caracterização dos custos relacionados à internação dos recém-nascidos prematuros, foram considerados somente os procedimentos que apresentaram valores aprovados na AIH, tanto os serviços hospitalares quanto profissionais. A caracterização dos custos foi descrita para cada grupo de prematuridade: prematuro extremo, moderado e tardio.

### Análise dos Dados

A caracterização dos custos foi realizada descritivamente, considerando-se a estratificação entre serviços hospitalares e profissionais. Além disso, foram referenciados, separadamente, os percentuais dos custos com base no valor total dos serviços hospitalares e profissionais. A incerteza dos parâmetros de custo (R$) foi estimada pelo intervalo de confiança de 95% (IC95%) e erro padrão (EP), por meio do método de Bootstrapping, com 1.000 amostragens.

Os dados de caracterização dos participantes foram apresentados descritivamente, estimando-se a média e o desvio-padrão para variáveis numéricas e medidas de frequência para variáveis categóricas.

Foi realizada uma análise de sensibilidade determinística, com base nos limites inferiores e superiores do intervalo de confiança de 95% de cada componente de custo (serviços profissionais e hospitalares), como parâmetros para as estimativas de variação nos custos (custo total e custo total/criança).

Realizou-se uma análise de regressão linear generalizada com distribuição *gamma*. Esse modelo foi adotado por permitir análise na qual as variáveis de resposta não apresentem distribuição normal. Além disto, o modelo possibilita inferências sobre a média de custos, o que o tornou adequado para este estudo. Para a definição das variáveis preditoras, foi verificada a colinearidade entre variáveis (estabelecida como r > 0,7). As variáveis colineares foram excluídas. Considerou-se como variáveis preditoras relacionadas ao nascimento dos bebês prematuros as variáveis idade gestacional, peso ao nascer, necessidade de suporte ventilatório (ventilação mecânica, ventilação não invasiva e oxigenioterapia) e necessidade de internação na UTIN. Como variáveis preditoras relacionadas as condições maternas foram considerados a idade materna, o número de consultas pré-natal e a presença, ou não, de riscos gestacionais (hipertensão, diabetes e infecção urinária). As variáveis de resposta analisadas foram os custos hospitalares e o tempo de internação.

A primeira análise de regressão teve como variável de resposta o custo total da internação. O modelo 1 verificou o impacto das variáveis maternas (idade materna, número de consultas pré-natal, presença de síndrome hipertensiva e infecção do trato urinário) e o modelo 2 verificou as variáveis do recém-nascidos prematuros (uso de ventilação mecânica, uso de oxigenoterapia, uso de ventilação não-invasiva, necessidade de internação em UTIN, idade gestacional e peso ao nascer) no custo total. A segunda análise de regressão considerou como variável de resposta o tempo de internação. O modelo 3 verificou os impactos das variáveis maternas (as mesmas apresentadas no modelo 1); e o modelo 4, as variáveis do recém-nascido (as mesmas citadas no modelo 2).

Foi adotado intervalo de confiança de 95% (IC95%), com significância de 5% (p < 0,05) e função link de identidade para estimativa do modelo. O critério de informação de Akaike foi utilizado para verificar o ajuste dos modelos, confirmando a distribuição *gamma* como a mais apropriada. A análise foi realizada por meio do programa SPSS, versão 25.

## RESULTADOS

Foram analisados os prontuários de todos os recém-nascidos no hospital investigado com registro de diárias de unidade de terapia intensiva neonatal e/ou diárias a mais em 2018 (totalizando 372 recém-nascidos elegíveis). Desses, foram excluídos 187 recém-nascidos a termo e 38 recém-nascidos prematuros com base nos critérios do estudo. Deste modo, foram incluídos 147 recém-nascidos prematuros em 2018.

A Tabela 1 apresenta os dados de caracterização dos participantes. Houve registro de 51 diárias de UTIN faturadas na AIH e 119 registros de diárias de permanência a mais. Os recém-nascidos que não foram internados na UTIN permaneceram internados nas demais unidades da neonatologia do hospital. Nesses casos, não foi possível identificar em qual unidade específica da neonatologia o recém-nascido ficou internado (esses dados estão referenciados na Tabela 1 como demais unidades da neonatologia). Destaca-se ainda que houve recém-nascidos que permaneceram internados tanto na UTIN quanto nas demais unidades da neonatologia.

**Tabela 1:** Dados de caracterização dos participantes, representados em frequência de ocorrência, média e desvio-padrão (quando aplicáveis).

Características	n	Frequência (%)	Média	DP
Idade gestacional (semanas)	147	-----	32,8	2,5
Peso ao nascer (gramas)	147	-----	1.792,0	526,0
Classificação geral dos recém-nascidos				
Adequado para idade gestacional	98	66,7	-----	-----
Pequeno para idade gestacional	48	32,7	-----	-----
Grande para idade gestacional	1	0,7		
Classificação da idade gestacional				
Extremo	4	2,7	25,7	1,3
Moderado	77	52,4	31,5	1,8
Tardio	66	44,9	34,8	0,8
Classificação do peso ao nascer				
Peso adequado	15	10,2	2.834	296,3
Baixo peso ao nascer	86	58,5	1.897,8	256,9
Muito baixo peso ao nascer	37	25,2	1.327,6	218,2
Extremo baixo peso ao nascer	9	6,1	954,4	193,8
Sexo				
Masculino	84	57,1	-----	-----
Feminino	62	42,2	-----	-----
Não identificado	1	0,7	-----	
Internação				
Somente na UTIN	51	34,7	18,6^a^	2,1
UTIN + demais unidades da neonatologia	119	81	9,6^b^	7,8
Somente demais unidades da neonatologia	96	65,3	8,94^c^	7,71
Suporte ventilatório				
Ventilação mecânica	37	25,2	-----	-----
Ventilação não invasiva^d^	90	61,2	-----	-----
Oxigenioterapia	87	59,2	-----	-----
Idade materna (anos)	147	------	26,3	7,4
Número de consultas pré-natal	147	------	5,6	3,7
Risco gestacional				
Diabetes	11	7,5	-----	-----
Síndrome hipertensiva	31	21,1	-----	-----
Infecção de trato urinário	52	35,4	-----	-----
Tipo de parto				
Normal	80	54,4	-----	-----
Cesáreo	67	45,6	-----	-----

^a,b,c^ Média (DP): média e desvio-padrão em dias de internação em: a) somente na unidade de terapia intensiva neonatal (UTIN); b) UTIN e nas demais unidades de neonatologia (unidades de cuidados intermediários convencional e canguru e/ou maternidade/alojamento conjunto); c) somente nas demais unidades de neonatologia descritas em b, à exceção da UTIN.

^d^ Ventilação não-invasiva: o bebê pode usar mais de uma modalidade de suporte.

Pode-se observar que a maioria foi composta por prematuros moderados, com peso adequado para a idade gestacional e classificados como baixo peso ao nascer. A predominância foi de parto normal e a maioria dos recém-nascidos foi do sexo masculino. A idade gestacional média foi de 32 semanas, com peso médio de 1.792 gramas. A maioria dos recém-nascidos necessitou de suporte de ventilação não-invasiva (Tabela 1).

A idade materna média foi de 26,2 anos e a média de consultas pré-natal foi de 5,6 consultas. Observou-se que a maioria das mães apresentou algum risco gestacional (Tabela 1).

Os custos totais advindos do nascimento e internação de recém-nascidos prematuros em 2018 foi de R$ 658.608,63. O custo total médio por recém-nascido extremo, moderado e tardio foi de R$ 17.395,46, R$ 6.688,92 e R$ 1.120,91, respectivamente. O custo médio por recém-nascido prematuro extremo foi 15,5 vezes e 2,6 vezes maior que recém-nascidos prematuro tardio e moderado, respectivamente. O custo médio por recém-nascido prematuro moderado foi de aproximadamente 6 vezes maior que o prematuro tardio (Tabela 2). A incerteza dos parâmetros de custo está apresentada na Tabela 3.

**Tabela 2:** Visão geral dos gastos com os componentes discriminados nas AIH, separados por serviços hospitalares e profissionais, além do valor do parto em cada um dos subgrupos de prematuridade. Foram referenciados os percentuais dos custos (%) ao valor total (serviços hospitalares e profissionais). Dados coletados em um hospital público de Brasília, DF, 2018.

Componentes	Pré-termo extremo (n = 4)		Pré-termo moderado (n = 77)		Pré-termo tardio (n = 66)		Total
	R$	%	R$	%	R$	%	
**Serviços hospitalares**							
Exames de imagem	179,98	0,26	2.463,82	0,48	1.755,98	2,37	4.399,78
Assistência fisioterapêutica	3.352,16	4,82	21.598,71	4,19	2.347,07	3,17	27.297,94
Terapia nutricional	1.080,00	1,55	15.225,00	2,96	810,00	1,09	17.115,00
Diárias	49.310,40	70,87	352.772,43	68,49	19.375,49	26,19	421.458,32
Tratamento de afecções originadas no período neonatal e doenças infecciosas	2.279,34	3,28	31.604,35	6,14	19.023,29	25,71	52.906,98
Outros	1.735,85	2,49	3.833,74	0,74	826,82	1,12	6.396,41
Custo do parto	1.300,33	1,87	26.803,22	5,20	2.3887,42	32,29	51.990,97
**Total dos serviços hospitalares**	**59.238,06**	**85,13**	**454.301,27**	**88,21**	**68.026,07**	**91,95**	**581.565,40**
**Serviços profissionais**							
Consultas médicas	198,52	0,29	5.225,70	1,01	3.074,91	4,16	8.499,13
Tratamento de afecções originadas no período neonatal	2.009,27	2,89	1.415,25	0,27	1.184,18	1,60	4.608,70
Diárias	8.136,00	11,69	54.104,40	10,50	1.695,00	2,29	63.935,40
Total dos serviços profissionais	10.343,79	14,87	60.745,35	11,79	5.954,09	8,05	77.043,23
**Custos totais**	**69.581,85**	**100,00**	**515.046,62**	**100,00**	**73.980,16**	**100,00**	**658.608,63**

AIH: autorização de internação hospitalar.

**Tabela 3:** Análise de incerteza dos parâmetros de custo (R$). As estimativas de intervalo de confiança de 95% e erro padrão foram realizadas por meio do método de Bootstrapping, com 1.000 amostragens.

	Média	EP	IC95%	
			Limite inferior	Limite superior
**Serviços hospitalares**				
Exames de imagem	29,93	2,64	24,72	35,02
Assistência fisioterapêutica	185,70	32,47	127,22	250,18
Terapia nutricional	116,42	14,04	89,79	144,88
Diárias	2.867,06	411,62	2.097,51	3.677,69
Tratamento de afecções originadas no período neonatal e doenças infecciosas	359,91	19,20	322,46	397,51
Outros	41,61	11,19	21,85	65,63
Custo do parto	353,68	5,77	342,33	365,02
**Total dos serviços hospitalares**	**3.954,33**	**460,21**	**3.084,50**	**4.887,49**
**Serviços profissionais**				
Consultas médicas	58,33	2,54	53,50	63,25
Tratamento de afecções originadas no período neonatal	18,10	1,20	16,23	20,70
Diárias	436,77	67,80	306,32	569,55
**Total dos serviços profissionais**	**513,22**	**68,15**	**382,48**	**647,81**
**Custos totais**	**4.467,55**	**527,98**	**3.469,43**	**5.537,15**

EP: erro padrão; IC95%: intervalo de confiança de 95%.

As despesas com serviços hospitalares foram 7,5 vezes maiores que os serviços profissionais. Mais de 70% das despesas totais corresponderam às diárias (somatório dos valores das diárias correspondentes aos serviços hospitalares e profissionais). As despesas com tratamento de afecções originadas no período neonatal e doenças infecciosas, despesas com o parto e assistência fisioterapêutica corresponderam a 8,7%, 7,9% e 4,14% dos gastos totais, respectivamente (Tabela 2).

O número de diárias de internação em UTIN nos prematuros extremo, moderado e tardio foi de 120 (4 recém-nascidos), 849 (77 recém-nascidos) e 44 (66 recém-nascidos), respectivamente. Tais diárias totalizaram um custo médio por recém-nascido de R$ 14.361,30 (extremo), R$ 5.284,11 (moderado) e R$ 319,92 (tardio). Os dados referentes à regressão estão apresentados na Tabela 4.

**Tabela 4:** Dados referentes à associação entre os fatores relacionados ao nascimento e às condições maternas e o custo e tempo de permanência hospitalar. Dados coletados em um hospital público de Brasília, DF, 2018.

Variáveis	Custo total (R$)			Permanência hospitalar (dias)		
	B^b^	IC95%	p	B^b^	IC95%	p
**Variáveis relacionados ao nascimento**						
**Constante**	5.624,40	1.782,10 a 9.466,60	-	87,21	66,79 a 107,63	-
Idade gestacional (semanas)	-123,00	-241,60 a -4,50	0,04	-2,20	-2,78 a -1,61	< 0,01
Peso ao nascer (gramas)	-0,20	-0,50 a 0,03	0,08	-0,03	-0,004 a -0,002	< 0,01
Internação em UTIN (sim ou não)^a^	6.932,70	5.309,40 a 8.556,00	< 0,01	1,67	-1,52 a 4,87	0,30
Uso de ventilação mecânica (sim ou não)^a^	736,90	-387,60 a 1.861,50	0,20	0,50	-3,52 a 4,51	0,81
Uso de ventilação não-invasiva (sim ou não)^a^	91,20	-156,90 a 339,40	0,47	3,04	1,14 a 4,92	< 0,01
Oxigenioterapia (sim ou não)^a^	21,40	-216,20 a 258,90	0,86	1,43	0,134 a 2,718	0,03
**Variáveis maternas**						
**Constante**	7.771,40	3.588,20 a 11.954,60	-	22,20	14,36 a 30,09	-
Idade materna (em anos)	-62,70	-190,20 a 64,80	0,33	-0,20	-0,48 a 0,02	0,07
Consultas pré-natal (quantidade)	-227,70	-403,30 a -52,00	0,01	-0,51	-1,13 a 0,12	0,11
Síndrome hipertensiva (sim ou não)^a^	1.820,00	-1.375,40 a 5.015,50	0,26	6,36	-0,16 a 12,88	0,06
Infecção trato urinário (sim ou não)^a^	-1.429,50	-3.444,40 a 585,30	0,16	0,14	-5,62 a 5,88	0,96

IC95%: intervalo de confiança de 95%; UTIN: unidade de terapia intensiva neonatal.

^a^ Ausência da condição (não) foi considerado categoria de referência.

^b^ Estimativa de parâmetro.

Verificou-se uma associação entre variáveis maternas e custos. Cada consulta de pré-natal realizada foi associada a uma redução de R$ 227,69 no custo total (Tabela 3). Houve associação entre custos e variáveis relacionadas ao nascimento, na qual a idade gestacional associou-se à redução de R$ 123,04 no custo total. Ademais, a necessidade de internação em UTIN foi associada a um aumento de R$ 6.932,70 no custo total (Tabela 4).

Quanto ao tempo de permanência, observou-se que a presença de síndrome hipertensiva foi associada a um aumento de 6 dias no tempo de permanência (Tabela 4). Quanto às variáveis relacionadas ao nascimento, quanto maior a idade gestacional, menor foi o tempo de permanência (Tabela 4). O uso de ventilação não-invasiva esteve associado ao aumento de 3 dias de internação e o uso de oxigenioterapia a 1 dia. O peso ao nascer contribuiu significantemente para o modelo.

A análise de sensibilidade identificou que os custos totais e os custos totais associados às diárias hospitalares, diárias do serviço profissional e assistência fisioterapêutica foram os componentes que mais influenciaram o modelo (Figura 1), indicando variações de aproximadamente 18%, 3% e 1,4%, respectivamente.

**Figura:** Análise de sensibilidade considerando as variações de parâmetros (limites inferiores e superiores do intervalo de confiança de 95%) de cada componente de custo (serviços profissionais e serviços hospitalares), considerando-se os custos totais (A e B) e custos totais/recém-nascido (C e D).

LI IC95%: limite inferior do intervalo de confiança de 95%; LS IC95%: limite superior do intervalo de confiança de 95%.

## DISCUSSÃO

O presente estudo demonstrou um custo médio de R$ 1.120 para prematuros tardios, R$ 6.688 para prematuros moderados e R$ 17.395 para prematuros extremos internados em um hospital público em 2018. Além disso, os fatores idade gestacional, internação em UTI neonatal e número de consultas pré-natal foram associados ao custo total. Além disso, os fatores presença de síndrome hipertensiva, idade gestacional, uso de ventilação não-invasiva e o uso de oxigenioterapia, foram associados ao tempo de permanência hospitalar.

Os custos totais com o nascimento prematuro foram de aproximadamente R$ 658 mil. Esse valor considerou despesas de apenas oito leitos da UTIN que são faturados no hospital investigado. Verificamos que a maior despesa ao considerar os componentes discriminados nas AIH foi referente às diárias de internação, as quais compreendem todas as ações necessárias para manutenção da vida, incluindo assistência profissional durante 24 horas ininterruptas. Ao analisarmos os custos de cada grupo de recém-nascidos, observamos que os recém-nascidos pré-termo extremos apresentam um custo médio por bebê de aproximadamente 3 vezes os moderados e 15 vezes os tardios.

A idade gestacional e o peso ao nascer se mostraram fatores importantes para os custos e tempo de permanência. Nesse sentido, os custos hospitalares foram maiores em recém-nascidos com menor idade gestacional. O aumento de cada semana de idade gestacional se associou a uma diminuição dos custos e do tempo de internação, enquanto o aumento de cada grama de peso ao nascer foi associado à diminuição do tempo de internação. Tais achados são corroborados por uma revisão sistemática^15^. Devido à imaturidade fisiológica, os recém-nascidos prematuros apresentam necessidade de suporte ventilatório, auxílio à alimentação e regulação de temperatura, sendo internados até atingirem o peso e os critérios para alta^8^. Tais aspectos contribuem para o aumento no tempo de internação e, consequentemente, a maior exposição a fatores estressores que impactam no desenvolvimento do recém-nascido^16^. Tais aspectos podem explicar o maior custo com as diárias de recém-nascidos extremos.

Quanto às condições clínicas dos recém-nascidos, observamos que a necessidade de internação na UTIN acarretou aumento nos custos. A necessidade de ventilação não-invasiva e oxigenioterapia associaram-se ao aumento no tempo de permanência. A necessidade de internação em UTIN foi associada a um aumento de aproximadamente R$ 6 mil no custo total. Estudos prévios também demonstraram custos elevados com a prematuridade, desde o nascimento até a internação em UTIN^5,9,17,18^. Essas descobertas corroboram nosso estudo e podem ser explicados pelo tipo de suporte e recursos oferecidos, como diárias, exames, medicamentos, oxigênio e honorários profissionais.

A realização de ventilação não-invasiva foi associada a um aumento de 3 dias de internação, enquanto o uso de oxigenioterapia acarretou aumento de 1 dia. Tais achados podem ser justificados pelas condições respiratórias ou condições pulmonares obstrutivas frequentes em recém-nascidos prematuros^8,18,19^. Estudos demonstraram que essas condições, tanto na internação neonatal quanto após a alta, bem como nos primeiros anos de vida, desencadeiam custos associados à internação e ao acompanhamento ambulatorial de recém-nascidos^8,19^. Crianças com quadros respiratórios podem necessitar de oxigenioterapia domiciliar ou, durante as crises, de acompanhamento e medicação. Ademais, um estudo prévio demonstrou que crianças com síndrome do desconforto respiratório tiveram custos mais elevados comparado a crianças sem a síndrome^20^, e que o custo com condições respiratórias representou 25% do custo total de internações de recém-nascidos prematuros^21^.

A presença de suporte ventilatório não foi associada a aumentos no custo da internação. Entretanto, consideramos relevante o fato de que o recém-nascido prematuro apresente imaturidade pulmonar e, ao nascer, é exposto a fatores que podem desencadear lesões, incluindo a ventilação mecânica^22^. O desmame da ventilação é lento e prolonga a internação, pois depende da resolução da lesão pulmonar ou de outras condições^23^. Assim, a utilização de ventilação mecânica influencia a permanência hospitalar e, consequentemente, deveria aumentar os custos. Entretanto, supomos que os custos não foram impactados, pois os valores de referência desse procedimento no SIGTAP são menores do que os praticados na atenção privada. Portanto, mudanças na perspectiva do estudo poderiam alterar os custos.

Demonstramos também que cada consulta adicional de pré-natal foi associada à redução dos custos. Essa redução pode estar relacionada à diminuição de complicações perinatais à medida que o pré-natal é realizado adequadamente^24,25^. Esse dado é relevante, pois a ausência de acompanhamento pré-natal pode aumentar em até 2,8 vezes o risco de prematuridade^26^. No Brasil, vários estudos demonstraram uma relação inversamente proporcional entre prematuridade e realização de pré-natal^24,25^. O pré-natal envolve diversas ações de prevenção e promoção da saúde, diagnóstico e tratamento, estruturadas em, no mínimo, seis consultas, sendo uma no primeiro trimestre de gestação, duas no segundo e três no terceiro^24^. Os dados de nossa pesquisa reforçam a importância do pré-natal, tendo em vista que os custos de atendimento de recém-nascidos das mães que não realizaram o pré-natal podem ser maiores quando comparado a recém-nascidos com pré-natal^27^. O acompanhamento pré-natal também é essencial para a assistência às mães que apresentam síndrome hipertensiva (fator de risco para a prematuridade e complicações gestacionais). A síndrome hipertensiva pode causar restrição do crescimento intrauterino, sofrimento fetal, baixo peso ao nascer e prematuridade^28^. Verificamos que a síndrome hipertensiva foi associada a um aumento de 6 dias da internação. Tais resultados podem ser explicados pelo fator de risco para complicações fetais imposto por essa condição, que pode causar anóxia^29^ e, consequentemente, demandar suporte respiratório. Gagliardi et al.^30^ também encontraram associação positiva entre hipertensão gestacional e o risco de problemas respiratórios agudos e crônicos em recém-nascidos.

Verificamos uma associação entre intervenções e condições relacionadas à prematuridade no contexto hospitalar. É importante elencar o fortalecimento da atenção primária à saúde, que pode auxiliar na redução dos custos com a atenção hospitalar no âmbito da prematuridade. Estudos prévios demonstraram que a ausência de acompanhamento pré-natal é fator de risco para o nascimento prematuro^24,25,27^. Ademais, verificamos uma associação entre a realização de consultas de pré-natal e o aumento do custo do recém-nascido prematuro. Assim, supomos que o aumento na cobertura e ampliação de ações para envolver a gestante na atenção pré-natal poderia prevenir a prematuridade e contribuir para a redução do impacto econômico dessa condição.

Vale destacar que nosso estudo considerou apenas a perspectiva do SUS. Entretanto, verificamos que o hospital investigado realizou procedimentos que não foram computados, por exemplo, a internação nas unidades de cuidados intermediários neonatais. Essas unidades não são credenciadas junto ao Ministério da Saúde e, assim, os custos dispendidos não foram incluídos na pesquisa. Contudo, a assistência ao recém-nascido prematuro nesses serviços foi considerada. Deste modo, é possível supor que os custos com a prematuridade sejam maiores. Trata-se de um achado relevante, pois o credenciamento das unidades de cuidados intermediários neonatais do hospital investigado poderia ampliar os recursos econômicos e os repasses do governo. Por fim, para colocar os custos da prematuridade sob perspectiva do hospital investigado, verificamos que os custos diretos advindos da assistência hospitalar a recém-nascidos prematuros foi considerável, uma vez que o custo médio de internação de um recém-nascido prematuro foi de R$ 4.480,33 enquanto o custo médio das internações pediátricas, considerando o tratamento das doenças crônicas das vias aéreas inferiores (doença mais prevalente no hospital) foi de R$ 548,03.

A análise de sensibilidade demonstrou que o valor das diárias hospitalares e diárias do serviço profissional foram os componentes que mais influenciaram o custo total estimado pelo nosso estudo. Tais achados indicam que as diárias hospitalares são importantes motivadores do modelo e apresentam uma maior determinação dos custos totais. Esse aspecto era esperado, considerando que as diárias de UTI são mais caras e, adicionalmente, esse setor demanda um cuidado especializado e de maior complexidade^31^. Cabe ressaltar que os dados da análise de sensibilidade foram baseados em variação ocorrida dentro de cada (AIH) analisada no estudo, que reflete a realidade de registro dos procedimentos usados pelos recém-nascidos prematuros de um hospital público. Em outros termos, não foi baseado em pressupostos arbitrários. Embora possa haver subnotificação de procedimentos realizados, percebe-se que não há uma variação substancial nos valores reembolsados pelo Ministério da Saúde aos hospitais para essa população investigada. Esse aspecto foi demonstrado pelas análises de sensibilidade univariadas, em que o parâmetro de diárias hospitalares determinou uma variação de 18% no custo total estimado.

Naturalmente, nosso estudo tem limitações, entre eles o número reduzido de participantes, pois, apesar do hospital investigado apresentar um alto índice de partos, não possui registros que permitam identificar todos os recém-nascidos prematuros. Todos os recém-nascidos prematuros que faturaram diárias foram incluídos, porém, a amostra não compreendeu todos os prematuros nascidos no hospital porque as unidades de cuidados intermediários do hospital não são credenciadas junto ao Ministério da Saúde e, consequentemente, não geram registro de diárias de neonatais nas AIH. Diante disso, prematuros que permaneceram internados apenas nessas unidades e não geraram custos com diárias não foram incluídos no estudo. Por esse motivo, tentamos minimizar o viés ao incluir todos os dados de custo referentes à internação de recém-nascidos prematuros disponíveis.

## CONCLUSÃO

Nossos achados demonstraram que os custos diretos advindos da assistência hospitalar a recém-nascidos prematuros em um hospital de grande porte, em 2018, foi de R$ 658.608. Verificamos que a prematuridade extrema impôs um custo 15,5 vezes maior comparado à prematuridade tardia. Ainda, verificamos que uma maior quantidade de consultas pré-natal e as semanas gestacionais foram associadas a uma redução dos custos da prematuridade.
